# Trends in the Binding of Cell Penetrating Peptides to siRNA: A Molecular Docking Study

**DOI:** 10.1155/2017/1059216

**Published:** 2017-02-21

**Authors:** P. V. G. M. Rathnayake, B. G. C. M. Gunathunge, P. N. Wimalasiri, D. N. Karunaratne, R. J. K. U. Ranatunga

**Affiliations:** ^1^Postgraduate Institute of Science, University of Peradeniya, 20400 Peradeniya, Sri Lanka; ^2^Department of Chemistry, University of Peradeniya, 20400 Peradeniya, Sri Lanka

## Abstract

The use of gene therapeutics, including short interfering RNA (siRNA), is limited by the lack of efficient delivery systems. An appealing approach to deliver gene therapeutics involves noncovalent complexation with cell penetrating peptides (CPPs) which are able to penetrate the cell membranes of mammals. Although a number of CPPs have been discovered, our understanding of their complexation and translocation of siRNA is as yet insufficient. Here, we report on computational studies comparing the binding affinities of CPPs with siRNA, considering a variety of CPPs. Specifically, seventeen CPPs from three different categories, cationic, amphipathic, and hydrophobic CPPs, were studied. Molecular mechanics were used to minimize structures, while molecular docking calculations were used to predict the orientation and favorability of sequentially binding multiple peptides to siRNA. Binding scores from docking calculations were highest for amphipathic peptides over cationic and hydrophobic peptides. Results indicate that initial complexation of peptides will likely occur along the major groove of the siRNA, driven by electrostatic interactions. Subsequent binding of CPPs is likely to occur in the minor groove and later on bind randomly, to siRNA or previously bound CPPs, through hydrophobic interactions. However, hydrophobic CPPs do not show this binding pattern. Ultimately binding yields a positively charged nanoparticle capable of noninvasive cellular import of therapeutic molecules.

## 1. Introduction

Recently, therapeutics have shown a vast diversification from small molecule drugs. Peptide and nucleic acid based therapeutics are among these alternatives and have been developed tremendously, to the point of clinical trials [1–3]. Short interfering RNA (siRNA) is among the therapeutics which have captured the interest of researchers [1, 4–7]. Once introduced into cells these oligonucleotides drive the RNA interference (RNAi) process [8], in which the expression of a target protein is suppressed by stimulating the specific degradation of messenger RNA. This mechanism leads to high specificity and large number of possible targets.

Major hurdles in the deployment of siRNA therapeutics are the low intracellular stability and low cellular uptake [9, 10]. A general cause of lower cellular uptake of gene therapeutics is the poor penetration through the cell membrane, which is efficient in regulating the internalization of foreign substances. Numerous carriers and drug delivery systems have been developed [11–14], including viral delivery, electroporation [15], and encapsulation and association of drugs with lipids [16], peptides [17–19], polymers [20], nanotubes [21], liposomes [22], micelles [23], and dendrimers [24].

This paper focuses on cell penetrating peptides (CPPs) with respect to their delivery of siRNA to cells through the use of computer modeling and simulation. We studied the structural features of seventeen CPPs and their capacity to form noncovalent complexes with siRNA.

### 1.1. siRNA and the RNA Interference (RNAi) Pathway

**Figure 1:**
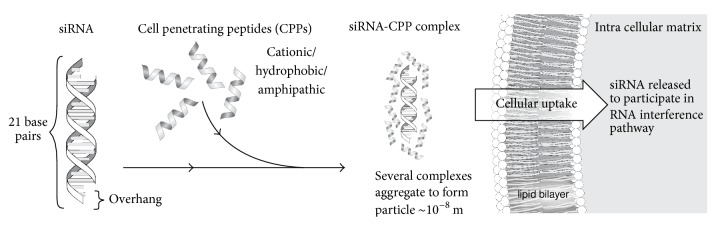
Cell penetrating peptides can bind with siRNA to form dense complexes. Several of those complexes can aggregate and translocate through the cell membrane.

Unlike most RNAs, siRNA is a double strand (ds) RNA with ~19–23 base pairs with characteristic 3′ overhangs (see Figure 1). Having overhangs facilitates the recognition by the enzymatic machinery of RNAi [4]. Usually, the siRNA is generated by Rnase III, the “Dicer” enzyme which cleaves long dsRNA into siRNA. The siRNAs can then bind to the RISC (RNA-induced silencing complex). Within the RISC, the siRNA is unwound and the sense strand is removed for degradation by nucleases present in the cell. The antisense strand specifically targets certain sequences of mRNA and directs them to the RISC. Then, it anneals with the complementary base pair. Finally, a rapid degradation of the target mRNA takes place, and consequently decreased protein expression results.

For siRNA to become a viable therapeutic, enzyme and environmental degradation have to be overcome with a proper delivery system. Therefore, extensive attention has been given to noninvasive peptide based delivery of siRNA into mammalian cells.

### 1.2. Cell Penetrating Peptides (CPPs)

Cell penetrating peptides are a distinct class of small peptidic molecules, having a special ability to trigger the movement of molecules across the cell membrane. CPPs have been shown to penetrate the cell membrane as well as mitochondrial and nuclear membranes without damaging them [25]. Known CPPs share many structural features and physical properties. Firstly, they are all water soluble. Secondly, CPPs are relatively small in size, not having more than 35 amino acid residues. Moreover, the cytotoxicities of these molecules are very low [26]. There is no unique classification for CPPs. They can be categorized according to their origin, according to their ability to link with the cargo, and according to their structure. Classification of CPPs by their structure separates peptides as (i) cationic CPPs, (ii) amphipathic CPPs, and (iii) hydrophobic CPPs.

### 1.3. CPP-siRNA Complexes

CPPs of all three classes have been used for the delivery of siRNA with success. Here several peptides adsorb on the surface of siRNA. Interactions driving peptide adsorption can be nucleotide specific, or nonspecific. Protein-nucleic acid complexation is primarily governed by electrostatic interactions where the negatively charged backbone of the polynucleotide is the key acceptor of charged species.

The secondary structure and shape complementarity also plays a role in the binding of peptides to nucleotides. Previously Schleif et al. speculated that helical peptides would show high binding affinities with double stranded RNA. The authors suggested that when the alpha-helix CPP is tilted, it can fit into the major groove of the dsRNA via hydrogen bonds and van der Waals interactions [27–29]. Furthermore, nitrogen base pairs which are exposed grooves of the RNA can become involved in sequence specific H bonds [28, 30].

**Figure 2:**
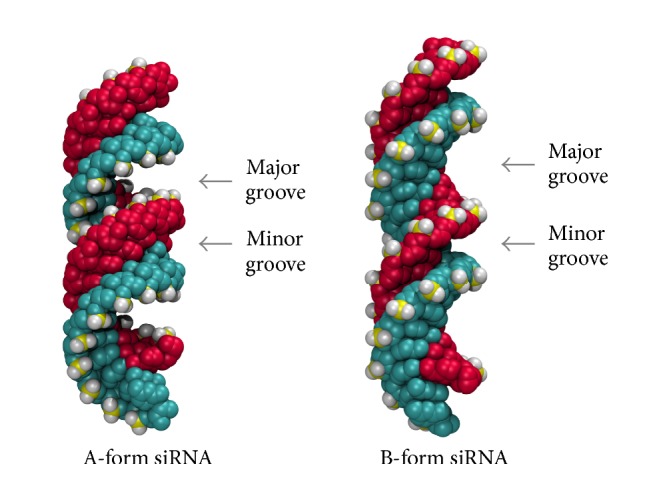
Structural differences of major groove and minor groove of A-form and B-form of siRNA (glyceraldehyde-3-phosphate dehydrogenase downregulating siRNA is represented here).

It follows that the conformation of dsRNA influences the binding of peptides due to the presence of 2′-OH groups in RNA; it naturally exists mostly in the thermodynamically stable A-form [31]. Thus DNA or B-formed RNA (see Figure 2) will not trigger the RNAi pathway. However, it is important to look into both conformations of RNA to get a broader idea about binding of CPPs to comprehend if there is a significant difference in between the bindings and thereby analyze any alternative means to fine-tune the siRNA-CPP complex.

As a result of inherent peptide-nucleic acid interaction, complexation of siRNA with multiple peptides usually occurs. This leads to the formation of complex, with a net positive charge. Further aggregation of these complexes forms dense nanoparticles, with a size of 10^2^ nm that may be internalized via endosomes [12, 32, 33]. This formation of a positively charged nanoparticle is important for translocation into the cell membrane and delivering the therapeutics. Here, our main focus is to study the formation of the positively charged complex which is the initial step of internalization [34].

In this research, we study the initial binding of CPPs to siRNA and focus on the interactions governing the complexation. To achieve a complete understanding about the binding of CPPs to siRNA, we performed docking calculations for 17 different peptides from all the structural classes. Calculations were performed for binding of CPPs for both A- and B-forms of the same GAPDH siRNA (see Figure 2). This siRNA downregulates the translation of glyceraldehyde-3-phosphate dehydrogenase (GAPDH) enzyme. GAPDH catalyses the conversion of glyceraldehyde-3-phosphate to D-glycerate 1,3-bisphosphate in the glycolytic breakdown of glucose. Furthermore, best scored CPPs from three categories were docked with another different siRNA, HPV si16E6, to confirm the binding pattern. To generalize the findings of the study, siRNA downregulating Human Papillomavirus (type 16) E6 oncoprotein, si16E6, was used for comparison.

## 2. Methodology

### 2.1. Generating Coordinates of siRNA

For the study, two siRNA molecules were arbitrarily chosen, namely, the GAPDH and si16E6. Ideal A- and B-forms of helical structure of the glyceraldehyde-3-phosphate dehydrogenase (GAPDH), with a sense strand 5′-GACGUAAACGGCCACAAGUUC-3′ and antisense strand of 5′-ACUUGUGGCCGUUUACGUCGC-3′, were generated using the make-na online server (NAB [35], Nucleic Acid Builder based application with Generalized Born, Poisson-Boltzmann, or 3D-RISM implicit solvent models). Ideal A-form of si16E6 with sense strand 5′-GCAACAGUUACUGCGACGUUU-3′ and an antisense strand of 5′-ACGUCGCAGUAACUGUUGCUU-3′ was also generated similarly with the same procedure. Once created, the stability of siRNA in aqueous medium was checked through molecular dynamics simulations using explicit (TIP3P) water at physiological temperature (310 K), for 24 ns using NAMD code and CHARMM v27 force field with time step of 2 fs/ts. The particle-particle mesh Ewald method was used for the long-ranged Coulombic interactions. A cutoff of 12 Å was used for the van der Waals interactions and short-ranged Coulombic interactions. Neighbor atom pair-lists were truncated at a distance of 13.5 Å and were updated every 10 time steps. RATTLE/SHAKE algorithms were used to constrain the bond length of the hydrogen-heavy atom bonds.

### 2.2. Generating Coordinates of Cell Penetrating Peptides

Generation and structure prediction of CPPs were carried out using PEP-FOLD [36–38] 2011 online server. All peptides were subjected to energy minimization via the NAMD code [39] and minimized structures were used for further calculations. For the minimization, the CHARMM v27 force field was used with the conjugate gradient minimization scheme.

### 2.3. Molecular Docking Calculations

Docking calculations were performed by the ClusPro [40, 41] online server with default (balanced weight) configurations. For docking simulations of CPP with siRNA, the siRNA was taken as the receptor while the CPP was regarded as the ligand. Docking calculations were submitted and the resulting output with minimum energy and highest clusters was taken for further calculations. These structures were minimized as before using the NAMD code [39] and GROMACS [42] v5 with the CHARMM [43] v27 force field and used for subsequent docking calculations. In the next step, the CPP-siRNA complex was taken as the receptor, and another CPP was taken as the ligand. This process was carried out up to 30 CPPs and the generated binding scores and coordinates were recorded. This docking process was carried out for GAPDH (A- and B-forms) and HPV si16E6 (A-forms). For A-form of GAPDH minimization was done using NAMD code and the rest were carried out in GROMACS version 5. From the amphipathic, cationic, and hydrophobic classes, the three best scoring CPPs were used and docking studies carried out. However, for HPV si16E6, docking was carried out for three best scoring CPPs in three groups.

A similar procedure was used to evaluate the dimerization docking energy scores, but here only two peptides were considered.

### 2.4. ClusPro Scoring Function

The energy function used in ClusPro represents shape complementarity, electrostatic, and desolvation contributions [70].

(1) E=Eshape+ω2Eelec+ω3Epair,Eshape=Eattr+ω1Erep,

where *E* denotes the docking score, while *E*_shape_, *E*_elec_, and *E*_pair_ denote shape complementarity, electrostatic, and desolvation contributions, respectively. The shape complementarity term *E*_shape_ accounts for both attractive (*E*_attr_) and repulsive (*E*_rep_) interactions. ClusPro provides four variations of energy functions by varying *ω*_1_, *ω*_2_, and *ω*_3_ coefficients. Docking scores reported use the “balanced” energy function, where the weightage of the components is

(2) $$\begin{array}{c} E=0.40E_{rep}-0.40E_{att}+600E_{elec}+1.00E_{DARS}. \end{array}$$

Both attraction and repulsion terms were considered in the energy function with the same weight. Electrostatic interactions were considered between the two proteins surrounded by solvent using simplified Generalized Born (GB) theory, with constant radii. More importantly, ClusPro introduces new structure-based, pairwise intermolecular potential DARS (Decoys as the Reference State). Detailed information on these terms can be found in Kozakov et al. (2006).

### 2.5. Explicit Solvent Molecular Dynamics Simulations

Both NAMD and GROMACS code were used for molecular dynamics simulations with a time step of 2 fs/ts. All the simulations of involving GAPDH B-form siRNA were carried out in NAMD, while simulations involving GAPDH A-form siRNA and siR16E6 siRNA were run in GROMACS. To maintain the same environment CHARMM v27 force field was used along with a cutoff of 12 Å for the van der Waals interactions and short-ranged Coulombic interactions, while the particle-particle mesh Ewald method was used for the long-ranged Coulombic interactions. Neighbor atom pair-lists were truncated at a distance of 13.5 Å and were updated every 10 time steps. CHARMM compatible TIP3P explicit waters were used for all simulations. To constrain the bond length of the hydrogen-heavy atom bonds the LINCS algorithm and RATTLE/SHAKE algorithms were used in GROMACS and NAMD, respectively.

## 3. Results and Discussion

In this study, we investigated the binding of cell penetrating peptides (CPPs) onto short interfering RNA (siRNA). Since this delivery route is under vigorous development, structural features of peptides which increase the effectivity of siRNA delivery are of prime interest. Specifically, discovering features which enhance binding of peptides to siRNA or enhance the cellular uptake would be advantageous in future design of de novo peptides.

### 3.1. Binding of CPPs to siRNA

As described in the methodology, docking calculations were performed to investigate the binding of CPPs to the siRNA, particularly as a function of siRNA : CPP ratio. Calculations were carried out through the ClusPro server, which uses a rigid body docking algorithm in generating the favorable orientations of docking, based on clustering. Docking scores are an approximate analog to binding free energies calculated by more thorough computational techniques such as molecular dynamics simulations. However, due to the expense of free energy calculations, and the number of different binding energies which are required to exhaustively analyze this problem, we have used docking as a realistic alternative. Furthermore, we focus more on the molecular geometries produced, and trends in the binding scores are observed.

To generalize our results, cell penetrating peptides over a wide range of physicochemical characters were studied: cationic, amphipathic, and hydrophobic. Details on the CPP used in the study are given in Table 1, including the amino acid sequences, trivial name, nominal charge, and nominal charge per residue.

**Figure 3:**
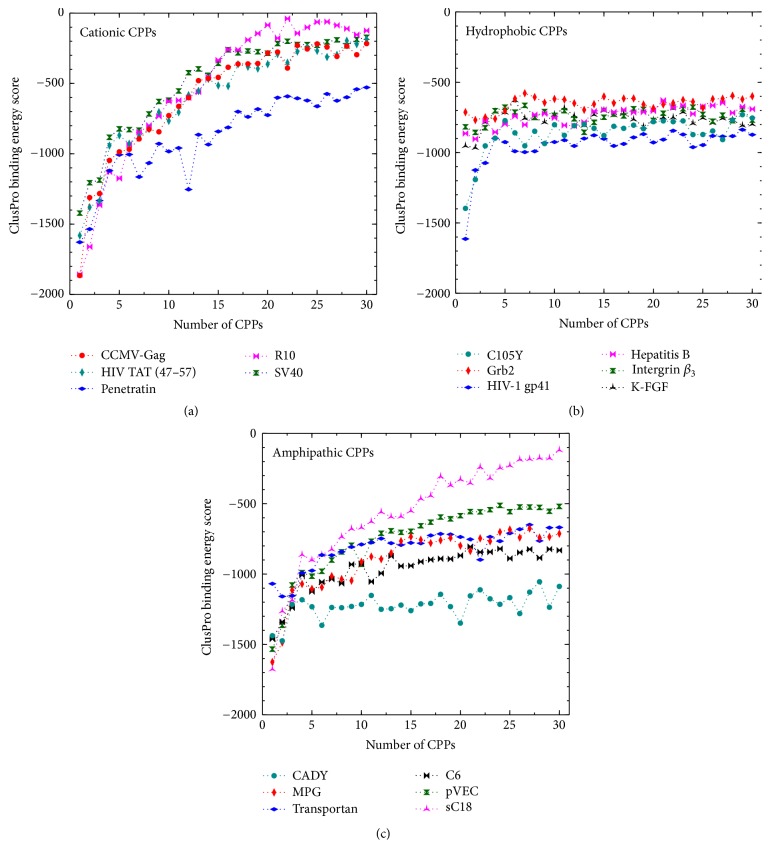
The variation in binding scores for the complexation of a successive number of peptides (see Table 1) with GAPDH (A-form).

**Figure 4:**
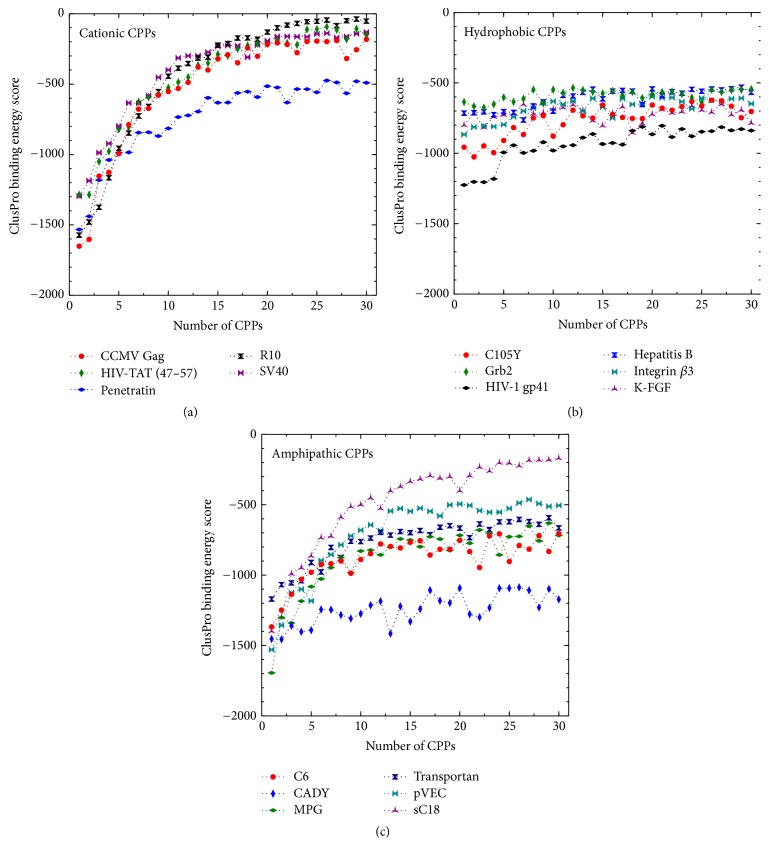
The variation in binding scores for the complexation of a successive number of peptides (see Table 1) with GAPDH (B-form).

The complete set of binding scores obtained by docking calculated are available in the supplementary data in Supplementary Material available online at https://doi.org/10.1155/2017/1059216. However, since we are more concerned about the trends in the sequential binding events rather than the absolute scores, a concise graphical representation of docking results is shown in Figures 3 and 4.

From the binding energies (shown in Figures 3 and 4) the general trend is apparent; initially the binding of CPP onto the siRNA is the most favorable, while the magnitude of the binding scores generally decreases with the number of CPPs complexed with the siRNA. To illustrate the trends specific to each class of peptide, we will discuss them separately.

*Cationic CPPs.* Cationic peptides are, by nature, rich in positively charged residues, mostly arginine and lysine. For example, R10 consists of only arginine. As a result of having these residues present, the number of possible intramolecular hydrogen bonds is decreased [71]. Therefore, the secondary structure presents predominantly coils and tubes, not helices [72]. The same phenomena can be seen with HIV-TAT (47–57), which is predicted to have a random coil structure.

All cationic peptides have a nominal charge between +7 and +10. Due to Coulombic attraction between the positively charged CPP and the negatively charged siRNA, peptides with higher positive charge density would bind more strongly. This is also supported by the binding scores for the complexation of the cationic peptides with siRNA (see Figures 3 and 4). Among the cationic peptides, R10 shows the maximum charge density (nominal charge density of 1.00) and consequently it displays relatively high affinity for the initial binding of peptides to the siRNA. Complexation driven by Coulombic interactions would be expected to decrease in magnitude due to progressively diminishing attractive force. This is illustrated through R10, which shows a drastic and monotonous lowering (in magnitude) of binding energy score at higher CPP : siRNA ratios, plateauing at around scores of −100 (Figures 3(a) and 4(a)). This trend is observed to a lesser extent in all of the cationic CPPs. Among them the highest gradient of binding energy score and largest initial binding energy score is displayed in CCMV-Gag and R10 with both forms of siRNA. Considering all cationic CPPs, it can be seen that Penetratin has low binding scores at high CPP : siRNA ratios. It stabilizes after binding of ~15th CPP at ~−700 binding score.

**Figure 5:**
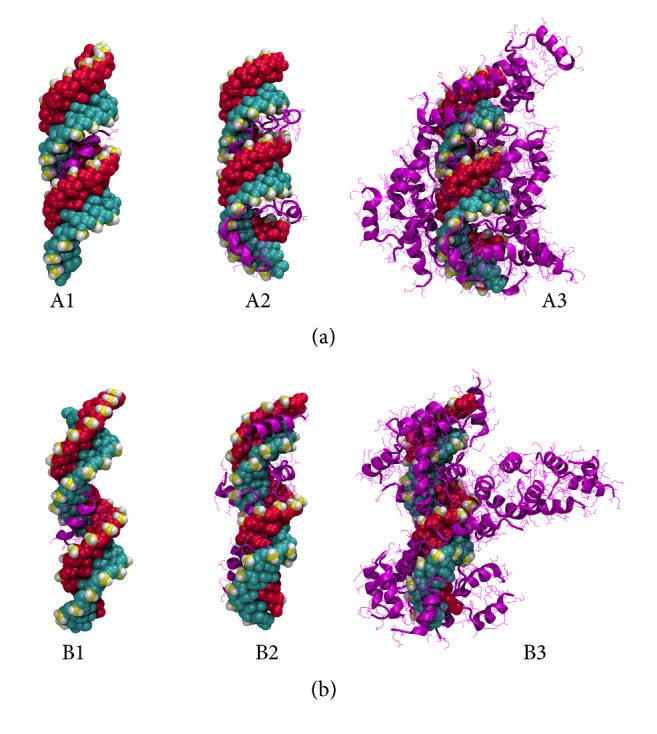
Predicted structures of CPP-GAPDH ((a) A-form; (b) B-form) complexes from docking calculations. Pictures show the Penetratin peptide representing the cationic class of CPPs. The peptide to siRNA ratios are 1 : 1 (left), 5 : 1 (center), and 30 : 1 (right), respectively.

When inspecting the orientation and placement of the cationic CPP on the siRNA (in both A- and B-forms), in all considered cases the initial CPP complexation occurs at the major groove of the siRNA (see Figures 5A1 and 5B1). However, unexpectedly, this is seen for both helical and random coil peptides. After the addition of a couple (two to three) of CPPs, the major groove is filled, and peptides preferentially bind to the minor groove of the siRNA, as seen in Figures 5A2 and 5B2. However, beyond 5–7 peptides bound (depending on the specific peptide), the peptides bind in a random orientation, seemingly driven by non-Coulombic forces.

At high CPP : siRNA ratios, the peptides seem not to bind directly onto the siRNA but rather interact with the peptide already bound onto the nucleotide. However, in the case of the cationic peptides, the ionic groups favor exposure to water and therefore the complex acquires a very open structure with high solvent accessible surface areas (SASAs) (see Figures 5A3 and 5B3). 

*Hydrophobic CPPs*. These peptides contain hydrophobic amino acid residues and display little or no charge at all. Due to the absence of charged residues, these peptides show diminished electrostatic interactions; binding to the siRNA is driven by van der Waals and hydrophobic forces. Consequently, the hydrophobic peptides show comparatively low magnitude in their binding scores, ranging from 600 to 1200. Furthermore, unlike the Coulombic interactions, the magnitudes of binding do not decrease in magnitude with the binding of CPP because the strength of hydrophobic and van der Waals interactions remains relatively constant. As a result, the binding scores of the hydrophobic peptides generally do not fall to values observed with the cationic peptide class.

**Figure 6:**
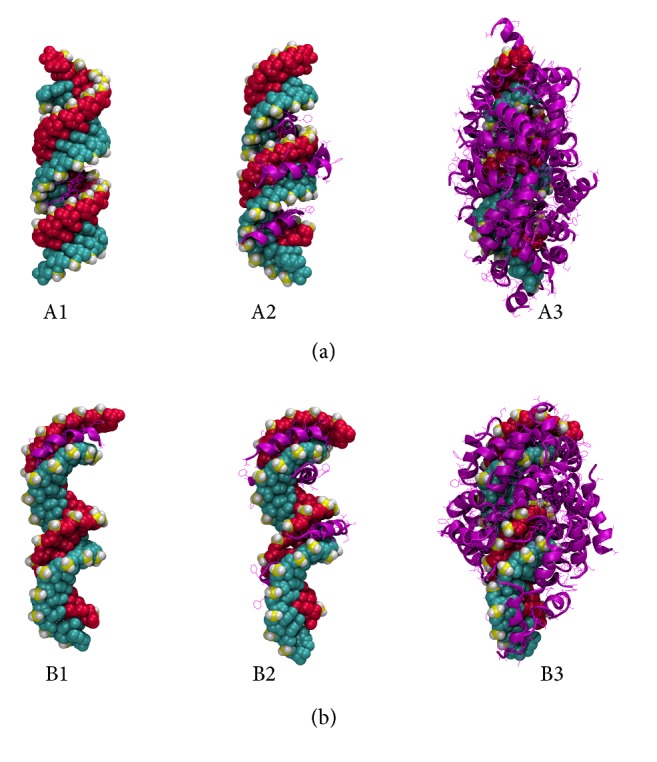
Predicted structures of CPP-GAPDH ((a) A-form; (b) B-form) complex from docking calculations. Pictures show the HIV-1 gp41 (1–23) peptide representing the hydrophobic class of CPPs. The peptide to siRNA ratios are 1 : 1 (left), 5 : 1 (center), and 30 : 1 (right), respectively.

Due to the weakness of van der Waals interactions, their total interactions tend to be nondirectional. Therefore, unlike cationic and amphipathic CPPs, the hydrophobic peptides do not follow specific orientations in binding to siRNA. Initially the hydrophobic peptides were predicted to bind to either the major groove or the minor groove. Figures 6B1 and 6B2 show the first two HIV-1 gp41 (1–23) peptides binding to the minor groove of the siRNA. However, in the A-form, HIV-1 gp41 (1–23) has randomly bound to the major groove, resulting in a significantly low binding energy score. Upon further binding of peptides, they show no orientational bias and complex in a random manner. Furthermore, because of the hydrophobic composition of the peptides, the peptide-siRNA complex arranges to minimize the surface area, as evidenced by the low solvent SASA values that were observed (see supplementary data). This leads to the formation of a dense, tightly packed peptide-siRNA particle (Figures 6A3 and 6B3). 

**Figure 7:**
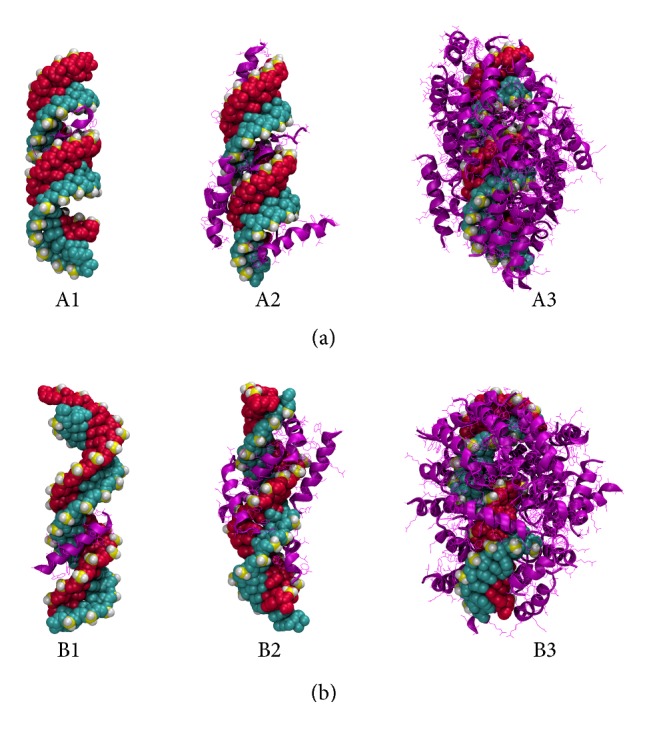
Predicted structures of CPP-GAPDH ((a) A-form; (b) B-form) complex from docking calculations. Pictures show the CADY peptide representing the amphipathic class of CPPs. The peptide to siRNA ratios are 1 : 1 (left), 5 : 1 (center), and 30 : 1 (right), respectively.

*Amphipathic CPPs.* These peptides exhibit both hydrophobic and hydrophilic behavior and/or regions in their structure. Usually, this capability comes with the presence of lysine as a component in the peptide [73]. All amphipathic peptides considered in the study are rich in lysine and have nominal charges between +4 and +8. Having both the positive charge of the cationic class and the nonpolar groups characterizing the hydrophobic peptides, this class shows the advantages of both. They show the initially strong binding of cationic peptides, a gradual decline, and finally plateauing (see Figures 3 and 4). As with the cationic CPPs, the amphipathic CPPs also bind initially to the major groove (Figures 7A1 and 7B1) with negative binding energy scores ranging from −1200 to −1800. However, when the number of CPPs is increased, binding energies rise sharply within 1–5 CPPs and becomes steady. Due to the presence of nonpolar groups, the hydrophobic effect allows the peptides to bind those peptides already present in the complex, resulting in relatively large binding energies at high CPP : siRNA ratios, compared to cationic peptides (see Figures 3(a), 4(a), 3(c), and 4(c)). Surprisingly, there is no clear trend between the predicted secondary structure (which is used in the docking) and the binding scores generated, where helical, beta-sheet, and random coil structures all exhibited similar binding scores. From the amphipathic peptides, sC18 and CADY indicate slight deviations. sC18 shows comparatively larger elevation of binding energies; CADY shows less. This indicates a favorable complexation of CADY and less favorable complexation of sC18 with siRNA.

Considering the results collectively, cell penetrating peptides with helical structures show higher binding affinity to siRNA such as CADY, C6, and sequence HIV-1 gp41 (1–23). By nature, most relatively small (<40 residue) peptides tend to arrange in helical structures; helices maximize intramolecular hydrogen bonding and provide better distribution of residues throughout the structure, which enables higher interacting power with the external environment. Spatial arrangement of cell penetrating peptides also provides less steric hindrance to bind to the major or the minor groove. Furthermore, having larger side chains on the peptide discourages the binding, thus showing higher binding energies. Particularly, peptides containing tryptophan and tyrosine exhibit slightly attenuated binding.

**Figure 8:**
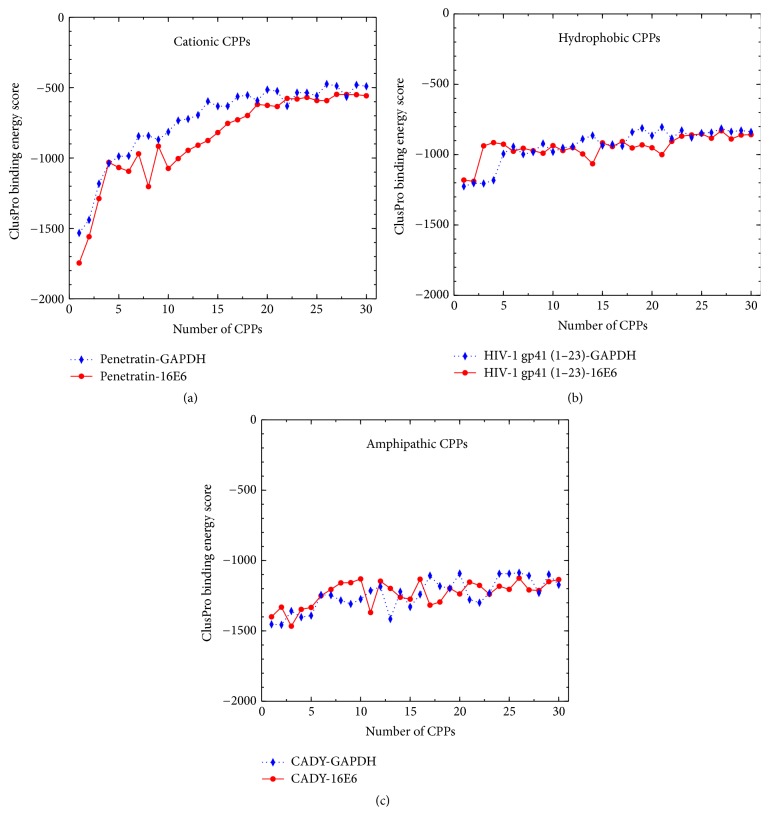
The variation in binding scores for the complexation of a successive number of cationic, hydrophobic, and amphipathic peptides with A-form of HPV-si16E6 and GAPDH siRNAs.

Since almost all siRNAs show identical structures, the same binding pattern was observed as shown in Figure 8. However, due to structural differences in major grooves and minor grooves, slight lowering of binding energy was observed in A-forms (for both GAPDH and HPV-si16E6).

Considering the results, the following stages of peptide binding can be extracted. (1) Initially electrostatic and shape complementarity drive the binding of cell penetrating peptides into the major groove of siRNA, where the maximum salt bridges may be formed. At this stage positive charge on the peptide is beneficial. Due to the small size of siRNA, only 2-3 CPPs can be accommodated into the major groove. (2) Subsequent peptides tend to bind to minor groove of siRNA while some get arranged perpendicular to the siRNA. Increased charge on the peptide tends to favor binding of the peptides to the minor groove. However, with the increasing number of peptides, more peptide-peptide interactions are observed. With further increase of the CPP to siRNA ratio, the peptides are observed to bind either onto the siRNA or to previously bound CPPs. Here, the competition of the peptide-siRNA and the peptide-peptide interaction play a major role. (3) Finally, once the surface charge on the siRNA has been screened, and a complete coating of the siRNA has taken place, further aggregation of peptides may occur, where the size of the siRNA-peptide complex is increased.

The docking scores for binding CPP initially to the A- and B-form show slight differences. The main reason that can be identified is the shape of the major groove of siRNA. The A-form helix is more coiled than the B-form helix, creating narrow, deep major groove and shallow, wide minor groove. Thus CPPs can interact with deeper major groove and thereby create a stable complex. This phenomenon is often evident in cationic CPPs since the positive charge drives Coulombic attraction with negative charges in nucleotide. Moreover, whenever a CPP is bound to major groove, it shows negatively larger binding score regardless of the type of CPP. One such example is initial binding of HIV1gp41 and C105Y to A-form of siRNA which is significantly lower than that of others since they are bound to major groove.

### 3.2. Aggregation and General Outlook

It goes without saying that the aggregation behavior of the peptides is essential information. To better evaluate this quantity, docking simulations of CPP dimerization were also carried out. Although approximate, the resultant scores agree with the expected trend; cationic peptides showed the lowest binding, while the amphipathic and hydrophobic peptides showed more favorable binding scores (see supplementary information for values). The balance between the peptide-siRNA and peptide-peptide interactions also affects the formation of nanoparticles and release of the therapeutic molecule(s) to the cytoplasm. Having a highly stable complex might be problematic when releasing the therapeutic molecule. On the other hand, if peptide-peptide interactions are too high, proteins will aggregate prior to complexing with siRNA. The size and nature of the aggregate are also important in the cell membrane translocation of siRNA-CPP complexes.

Experimental results suggest that aggregates of several siRNA-CPP complexes will interact with each other and produce larger particles (which reside in the 10^2^ nm size scale), which have the ability to overcome the cell membrane barrier.

Here, the siRNA : CPP 1 : 30 complexes are ~10 nm in size. However, among the mentioned three categories, a significant variation in the SASA can be seen (see supplementary information). For the cationic peptides, for siRNA : CPP of 1 : 30, the SASA values are in the range of 402–550 nm^2^, while for the hydrophobic and amphipathic peptide the ranges were 185–265 nm^2^ and 395–472 nm^2^, respectively. The size and the physicochemical nature of the exposed surface and the size of final multicomplex nanoparticle are known to have a large impact on the internalization. However, we cannot predict the final size of the aggregates using the techniques used in this research but only suggest that the highly charged complexes are unlikely to aggregate in large numbers due to the unfavorable charge and solvation effects.

The structures and associated binding scores calculated here should only be taken as representative. Although the docking algorithm used samples over many of the possible locations and orientations of binding, only limited flexibility is allowed for the peptide when binding. Therefore, firstly the initial predicted structure plays a large role in the predictions, and secondly the process does not sample the free energy of the binding processes with the correct weights. So there will be a significant error in the associated results. Given this limitation, we have taken care not to use the absolute binding scores in interpreting the results and use only the relative differences between the peptides, and the variation in binding scores for a single type of peptide, to minimize systematic errors. However, due to the sensitivity of peptide structure to environmental conditions, this is a large source of uncertainty in the study. We hope future studies of chosen peptides and structures may allow more quantitative information about the binding process, which will allow future experimentalists and peptide designers to further progress in this field. Because much of the mechanisms involved in this delivery mechanism are not fully understood, our knowledge in properties which enhance cellular uptake is largely empirical. More pedagogical investigation of each step in the pathway of complexation, internalization, release, and metabolism is required to fully understand and manipulate this attractive technology.

## Supplementary Material

Supplementary data includes amino acid sequence and secondary structure of cell penetrating peptides (CPPs) with their charge. Furthermore, binding energies with siRNA, dimerization energy, and SASA are included.

## Figures and Tables

**Table 1:** List of CPPs included in the study.

CPP type	CPP name	Amino acid sequence (underlined residues are positively charged)	Residue count	Nominal charge (charge per residue)
Cationic cell penetrating peptides	Penetratin [44, 45]	RQIKIWFQNRRMKWKK	16	+7 (0.44)
	HIV-TAT (47–57) [46, 47]	YGRKKRRQRRR	11	+8 (0.72)
	R10 [48, 49]	RRRRRRRRRR	10	+10 (1.00)
	CCMV Gag (7–25) [49–51]	KLTRAQRRAAARKNKRNTRGC	21	+9 (0.43)
	Chimeric dermaseptin S4 and SV40 ‘S413-PV' [11, 34]	ALWKTLLKKVLKAPKKKRKVC	21	+9 (0.43)

Amphipathic cell penetrating peptides	Transportan [52]	GWTLNSAGYLLGKINLKALAALAKKIL	27	+4 (0.15)
	pVEC (vascular endothelial cadherin) [53–56]	LLIILRRRIRKQAHAHSK	18	+6 (0.33)
	MPG [57, 58]	GALFLGFLGAAGSTMGAWSQPKKKRKV	27	+5 (0.16)
	CADY [59, 60]	GLWRALWRLLRSLWRLLWRA	20	+5 (0.25)
	sC18 [61]	GLRKRLRKFRNKIKEK	16	+8 (0.50)
	C6 [62]	RLLRLLLRLWRRLLRLLR	18	+7 (0.39)

Hydrophobic cell penetrating peptides	K-FGF (Kaposi's sarcoma fibroblast growth factor) [63]	AAVALLPAVLLALLAP	16	0 (0.00)
	Integrin *β*3-fragment [64, 65]	VTVLAGALAGVGVG	14	0 (0.00)
	Hepatitis B virus translocation motif [66]	PLSSIFSRIGDP	12	0 (0.00)
	Grb2 (SH_2_ domain) [67]	AAVLLPVLLAAP	12	0 (0.00)
	Fusion sequence HIV-1 gp41(1–23) [46, 68]	GALFLGFLGAAGSTMGA	17	0 (0.00)
	C105Y [69]	CSIPPEVKFNKPFVYLI	17	+1 (0.06)
