# Supplementary material for: Trends in the Binding of Cell Penetrating Peptides to siRNA: A Molecular Docking Study
Source: J Biophys. 2017 Feb 21;2017:1059216. doi: 10.1155/2017/1059216 (PMC5340175; doi:10.1155/2017/1059216)
Supplement: Supplementary file 1 — Supplementary data includes amino acid sequence and secondary structure of cell penetrating peptides (CPPs) with their charge. Furthermore, binding energies with siRNA, dimerization energy, and SASA are included. [file 1059216.f1.pdf]

Supporting information for
Trends in the binding of cell penetrating peptides to siRNA: A molecular docking study

P. V. G. M. Rathnayake[a], B. G. C. M. Gunathunge[b], P.N. Wimalasiri[b], D. N. Karunaratne[a,b], R. J. K. U. Ranatunga[a,b*]

[a]Postgraduate Institute of Science, University of Peradeniya, Peradeniya 20400, Sri Lanka
[b]Department of Chemistry, University of Peradeniya, Peradeniya 20400, Sri Lanka
*Corresponding author: udyranatunga@pdn.ac.lk

Supplementary data includes amino acid sequence and secondary structure of Cell Penetrating Peptides (CPPs) with their charge. Further, binding energies with siRNA, dimerization energy and SASA are included.

Basic information and Binding Energy Scores: Cationic Cell Penetrating Peptides with GAPDH (A-form and B-form) and HPV16E6

	**Penetratin**			**HIV-TAT (47–57)**		**R10**		**CCMV Gag**		**Chimeric dermaseptin S4 and SV40 ‘S413-PV’**	
	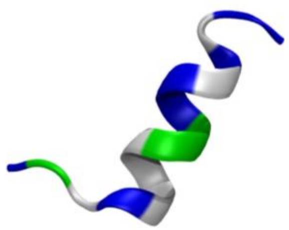			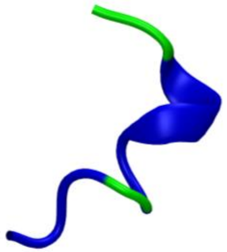		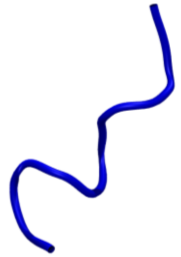		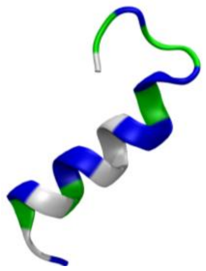		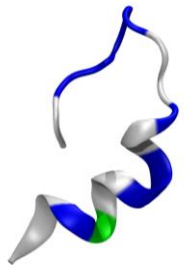	
Amino Acid Sequence	RQIKIWFQNRRMKWKK			YGRKKRRQRRR		RRRRRRRRRR		KLTRAQRRAAARKNKRN TRGC		ALWKTLLKKVLKAPKKKR KVC	
Charge	+7.00			+8.00		+10.00		+9.00		+9.00	
Residue count	16			11		10		21		21	
SASA	21.81 nm^2			17.92 nm^2		20.95 nm^2		24.72 nm^2		24.59 nm^2	
Dimerization energy Score	-642.1			-175.9		-138.5		-189.0		-174.7	
CPP count on complex	Binding Energy Score			Binding Energy Score		Binding Energy Score		Binding Energy Score		Binding Energy Score	
	GAPDH B-form	GAPDH A-form	HPV16E6 A-form	GAPDH B-form	GAPDH A-form	GAPDH B-form	GAPDH A-form	GAPDH B-form	GAPDH A-form	GAPDH B-form	GAPDH A-form
1	-1532.6	-1628.7	-1745.80	-1285.3	-1581.4	-1572.8	-1858.70	-1651.2	-1865.60	-1295.2	-1421.70
2	-1439.6	-1535.6	-1559.10	-1285.3	-1380.0	-1480.3	-1661.30	-1602.4	-1311.30	-1186.0	-1205.60
3	-1182.1	-1332.8	-1288.20	-1050.6	-1334.9	-1375.1	-1364.20	-1153.5	-1283.10	-986.6	-1187.10
4	-1038.8	-1120.4	-1031.10	-978.0	-941.7	-1165.3	-1128.10	-1126.3	-1047.40	-922.3	-882.80
5	-988.5	-1007.2	-1067.60	-821.3	-868.7	-956.3	-1175.30	-993.7	-986.10	-798.1	-823.10
6	-985.1	-1004.8	-1094.10	-804.4	-928.6	-847.5	-934.90	-788.4	-968.20	-633.9	-828.10
7	-844.8	-1164.9	-970.10	-626.3	-875.6	-726.0	-846.90	-677.6	-894.50	-636.2	-831.90
8	-842.0	-1065.7	-1203.00	-592.8	-824.4	-658.4	-798.40	-672.3	-825.80	-582.0	-716.70
9	-869.1	-928.6	-915.90	-571.6	-704.5	-553.7	-733.30	-578.3	-843.90	-452.3	-627.90
10	-814.5	-983.1	-1073.90	-525.2	-766.2	-444.0	-625.90	-552.7	-728.60	-399.1	-618.70

11	-734.0	-958.9	-1004.20	-486.3	-704.2	-386.8	-620.50	-531.0	-662.90	-314.2	-553.20
12	-722.4	-1253.8	-945.50	-450.8	-583.2	-354.0	-602.00	-487.2	-601.40	-299.1	-423.20
13	-695.1	-864.4	-909.10	-366.1	-553.8	-315.2	-560.30	-379.1	-480.00	-299.1	-396.70
14	-597.1	-934.2	-875.70	-351.4	-467.5	-308.5	-455.30	-399.8	-465.30	-273.4	-442.00
15	-632.0	-841.6	-819.00	-288.2	-514.7	-223.3	-334.50	-321.0	-456.90	-231.8	-356.80
16	-631.1	-812.7	-754.10	-298.8	-518.7	-211.6	-259.40	-291.5	-384.90	-226.1	-260.80
17	-562.8	-700.6	-728.50	-249.5	-364.3	-172.8	-260.90	-348.6	-361.80	-227.5	-282.20
18	-553.8	-737.5	-698.10	-225.8	-381.6	-171.6	-190.40	-243.5	-361.00	-308.9	-269.10
19	-592.1	-682.7	-619.40	-213.8	-394.5	-181.4	-144.80	-302.3	-358.40	-220.6	-274.60
20	-514.6	-725.3	-626.00	-213.1	-362.7	-130.5	-85.10	-217.9	-283.20	-193.2	-285.20
21	-524.3	-600.9	-634.20	-187.1	-290.6	-100.5	-178.30	-207.1	-276.70	-162.3	-216.20
22	-630.9	-591.7	-576.70	-208.4	-352.4	-81.0	-39.20	-218.3	-390.60	-161.4	-200.30
23	-535.4	-605.8	-580.70	-220.8	-273.5	-69.3	-143.40	-276.9	-229.20	-163.2	-222.90
24	-535.8	-621.4	-569.20	-111.5	-243.2	-56.8	-101.30	-197.7	-254.90	-163.8	-223.70
25	-557.9	-662.4	-591.50	-109.6	-268.1	-53.8	-63.30	-194.5	-218.90	-143.7	-228.60
26	-474.4	-574.5	-592.30	-93.2	-312.3	-44.9	-61.10	-197.8	-241.30	-139.0	-204.10
27	-488.3	-622.7	-547.70	-114.3	-296.7	-85.0	-87.00	-190.7	-308.20	-170.0	-190.40
28	-565.6	-598.2	-548.40	-181.2	-196.3	-49.5	-110.70	-318.2	-235.20	-162.1	-231.90
29	-480.1	-541.2	-550.10	-106.3	-221.0	-38.0	-153.50	-255.6	-295.70	-142.6	-187.30
30	-490.5	-528.5	-557.90	-151.3	-186.9	-52.3	-124.50	-181.9	-216.60	-130.7	-171.90
∑	**-22054.9**	**-26230.8**	**-25677.10**	**-13185.3**	**-17692.2**	**-12866.2**	**-15702.50**	**-15557.0**	**-17837.60**	**-12024.4**	**-14764.70**

Basic information and Binding Energy Scores: Hydrophobic Cell Penetrating Peptides with GAPDH (A-form and B-form) and HPV16E6

	**K-FGF Kaposis sarcoma fibroblast growth factor**		**Integrin β3-fragment**		**Hepatitis B virus translocation motif**		**Grb2 (SH2 Domain)**		**Fusion sequence HIV-1 gp41(1–23)**			**C105Y**	
	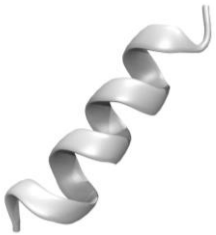		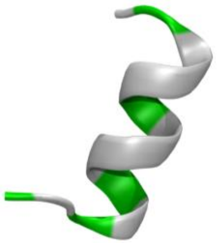		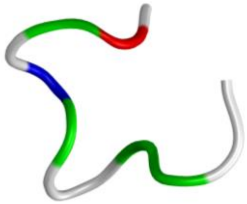		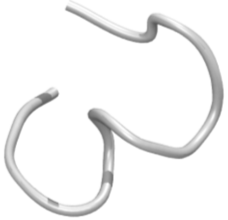		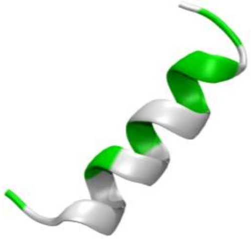			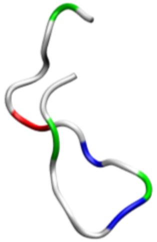	
Amino Acid Sequence	AAVALLPAVLLALLAP		VTVLAGALAGVGVG		PLSSIFSRIGDP		AAVLLPVLLAAP		GALFLGFLGAAGSTMGA			CSIPPEVKFNKPFVYLI	
Charge	+1.27e-7		+8.94e-8		+7.08e-8		+9.69e-8		+1.23e-7			+1.00	
Residue count	16		14		12		12		17			17	
SASA	18.19 nm^2		13.21 nm^2		13.36 nm^2		12.02 nm^2		16.31 nm^2			18.19 nm^2	
Dimerization Energy Score	-360.5		-376.6		-282.0		-292.4		-517.7			-513.7	
CPP count on complex	Binding Energy Score		Binding Energy Score		Binding Energy Score		Binding Energy Score		Binding Energy Score			Binding Energy Score	
	GAPDH B-form	GAPDH A-form	GAPDH B-form	GAPDH A-form	GAPDH B-form	GAPDH A-form	GAPDH B-form	GAPDH A-form	GAPDH B-form	GAPDH A-form	HPV16E6 A-form	GAPDH B-form	GAPDH A-form
1	-800.2	-952.10	-866.9	-816.90	-714.8	-864.00	-636.2	-712.70	-1225.3	-1613.80	-1180.40	-956.8	-1396.60
2	-716.9	-965.20	-813.6	-856.30	-712.2	-903.60	-666.4	-768.70	-1203.1	-1124.30	-1189.40	-1025.9	-1191.90
3	-816.5	-784.40	-805.1	-823.50	-706.0	-775.70	-675.5	-749.50	-1204.8	-1074.10	-937.40	-947.5	-952.20
4	-741.1	-709.70	-809.9	-702.10	-725.1	-854.50	-651.8	-759.90	-1182.0	-898.00	-914.50	-995.7	-897.50
5	-727.1	-714.10	-796.4	-677.30	-704.2	-796.30	-603.4	-703.30	-994.1	-924.90	-925.80	-909.1	-772.90
6	-717.9	-626.80	-741.4	-712.80	-709.5	-740.60	-635.0	-617.30	-943.2	-990.50	-975.80	-817.2	-859.70
7	-652.3	-756.30	-699.9	-663.90	-764.1	-802.10	-612.0	-578.40	-996.9	-996.60	-954.60	-867.0	-951.90

8	-719.2	-753.70	-679.3	-750.30	-663.6	-731.00	-548.0	-605.80	-982.4	-990.80	-972.20	-749.1	-848.30
9	-711.0	-783.80	-641.5	-708.10	-634.4	-723.40	-645.1	-645.20	-921.1	-930.80	-989.70	-733.1	-935.90
10	-686.5	-729.00	-631.0	-735.60	-701.3	-752.20	-547.9	-618.40	-980.5	-924.90	-935.90	-878.4	-802.00
11	-674.8	-683.40	-651.8	-702.20	-588.4	-805.80	-570.7	-623.20	-950.9	-911.50	-970.90	-797.6	-877.20
12	-655.7	-738.70	-627.8	-759.50	-593.0	-809.70	-536.0	-648.90	-942.4	-952.70	-950.00	-693.7	-808.60
13	-696.3	-807.10	-702.0	-856.00	-567.1	-784.90	-551.2	-696.30	-888.8	-900.20	-994.90	-734.3	-799.40
14	-768.3	-730.20	-610.2	-784.40	-544.4	-706.70	-563.5	-655.40	-863.5	-877.20	-1064.60	-750.1	-827.50
15	-803.8	-700.10	-672.2	-748.60	-613.4	-695.40	-575.0	-601.20	-934.5	-901.90	-916.10	-656.7	-878.60
16	-717.8	-733.30	-749.7	-726.60	-558.7	-712.20	-556.9	-648.50	-926.9	-952.30	-942.40	-723.8	-813.10
17	-668.8	-722.20	-610.8	-738.90	-553.8	-696.00	-588.5	-615.30	-938.6	-938.50	-906.70	-745.2	-827.70
18	-854.1	-763.20	-652.6	-688.40	-558.4	-713.70	-564.0	-614.60	-840.0	-892.10	-952.20	-752.6	-805.20
19	-787.4	-814.70	-650.9	-692.50	-654.0	-711.60	-608.6	-658.10	-811.8	-868.50	-930.60	-754.5	-830.20
20	-722.9	-769.30	-597.2	-771.50	-543.2	-703.00	-580.6	-683.90	-865.0	-927.80	-951.00	-657.2	-780.20
21	-689.5	-750.00	-606.0	-720.10	-590.0	-628.70	-566.0	-657.80	-804.4	-907.70	-999.80	-680.2	-774.80
22	-713.1	-750.00	-604.3	-677.00	-563.0	-679.70	-562.0	-647.70	-884.5	-844.30	-905.40	-694.1	-780.30
23	-705.2	-711.30	-633.6	-688.20	-573.7	-665.60	-579.3	-625.60	-827.8	-871.20	-868.60	-668.3	-774.70
24	-663.0	-791.90	-683.8	-676.80	-546.2	-724.50	-604.3	-638.90	-878.9	-961.40	-860.10	-632.1	-872.10
25	-693.9	-753.20	-608.2	-728.00	-559.1	-672.60	-640.6	-682.00	-846.6	-946.50	-852.50	-664.4	-871.50
26	-709.0	-788.80	-623.7	-777.10	-543.4	-664.90	-548.9	-618.80	-842.9	-881.40	-883.20	-623.1	-846.10
27	-650.8	-756.00	-631.2	-733.90	-550.1	-646.10	-565.2	-616.80	-814.4	-885.00	-830.40	-627.5	-907.70
28	-725.8	-767.90	-613.0	-773.10	-541.1	-717.40	-550.5	-595.80	-837.1	-882.90	-888.60	-665.5	-768.40
29	-693.0	-806.80	-609.0	-691.50	-530.5	-673.90	-548.2	-620.90	-828.2	-837.20	-860.40	-747.5	-730.50
30	-785.4	-797.60	-647.6	-769.40	-566.7	-690.80	-544.2	-599.30	-838.6	-872.90	-858.00	-703.2	-754.50
∑	**-21667.3**	**-22910.80**	**-20270.6**	**-22150.50**	**-18373.4**	**-22046.60**	**-17625.5**	**-19508.20**	**-27999.2**	**-28481.90**	**-28362.10**	**-22851.4**	**-25937.20**

Basic information and Binding Energy Scores: Amphipathic Cell Penetrating Peptides with GAPDH (A-form and B-form) and HPV16E6

	**Transportan**		**pVEC** **(Vascular endothelial cadherin)**		**MPG**		**CADY**			**sC18**		**C6**	
	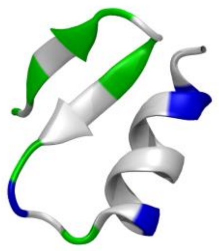		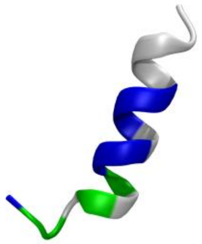		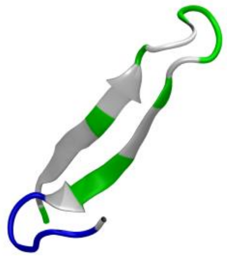		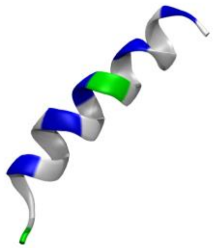			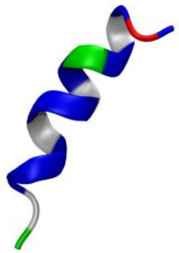		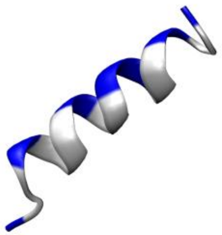	
Amino Acid Sequence	GWTLNSAGYLLGKIN LKALAALAKKIL		LLIILRRRIRKQAHAH SK		GALFLGFLGAAGST MGAWSQPKKKRKV		GLWRALWRLLRSLWRLLWRA			GLRKRLRKFRNKIKE K		RLLRLLLRLWRRLLRL LR	
Charge	+4.00		+6.00		+5.00		+5.00			+8.00		+7.00	
Residue count	27		18		27		20			16		18	
SASA	27.44 nm^2		23.16 nm^2		26.88 nm^2		25.02 nm^2			23.03 nm^2		25.19 nm^2	
Dimerization Energy Score	-529.7		-281.4		-564.2		-848.3			-286.3		-588.1	
CPP count on complex	Binding Energy Score		Binding Energy Score		Binding Energy Score		Binding Energy Score			Binding Energy Score		Binding Energy Score	
	GAPDH B-form	GAPDH A-form	GAPDH B-form	GAPDH A-form	GAPDH B-form	GAPDH A-form	GAPDH B-form	GAPDH A-form	HPV16E6 A-form	GAPDH B-form	GAPDH A-form	GAPDH B-form	GAPDH A-form
1	-1169.9	-1068.80	-1529.5	-1533.60	-1694.2	-1625.20	-1453.4	-1437.90	-1399.70	-1398.4	-1675.90	-1367.4	-1460.70
2	-1067.5	-1158.90	-1356.3	-1363.70	-1301.4	-1486.50	-1456.4	-1474.50	-1331.10	-1299.3	-1263.80	-1248.5	-1340.20
3	-1055.4	-1154.60	-1125.2	-1078.20	-1340.1	-1114.40	-1358.8	-1215.50	-1466.10	-991.5	-1185.20	-1136.5	-1239.20
4	-1039.8	-992.20	-1100.4	-990.20	-1184.8	-1070.50	-1402.5	-1182.60	-1346.90	-948.0	-862.50	-1030.0	-1007.20
5	-910.0	-975.60	-1183.8	-1015.10	-1082.0	-1105.10	-1391.0	-1232.30	-1333.10	-864.1	-900.10	-979.8	-1124.10
6	-977.6	-864.60	-895.7	-980.30	-1027.1	-1093.70	-1244.0	-1364.20	-1249.80	-733.9	-863.90	-924.8	-1056.80
7	-803.4	-866.50	-854.3	-900.30	-946.3	-1014.90	-1245.8	-1237.30	-1205.20	-724.2	-824.50	-919.1	-1035.40
8	-876.8	-841.70	-785.3	-842.50	-874.7	-1035.40	-1283.3	-1239.00	-1158.50	-589.1	-735.00	-899.6	-1066.30

9	-759.8	-807.30	-721.3	-795.40	-983.5	-1046.90	-1308.2	-1230.00	-1157.00	-513.4	-677.80	-987.1	-931.50
10	-762.0	-789.60	-681.3	-931.00	-829.1	-913.00	-1274.3	-1215.60	-1130.50	-500.0	-669.20	-888.8	-924.90
11	-736.8	-775.20	-643.2	-766.70	-822.7	-876.40	-1214.2	-1152.00	-1369.00	-451.2	-627.00	-847.6	-1054.20
12	-696.1	-747.70	-684.3	-710.20	-856.0	-894.20	-1185.2	-1250.60	-1146.40	-526.0	-558.70	-778.5	-994.80
13	-715.6	-780.50	-544.3	-692.70	-802.0	-851.30	-1415.4	-1246.10	-1198.00	-401.1	-592.70	-796.1	-871.70
14	-690.5	-793.10	-527.1	-703.50	-742.9	-765.90	-1221.1	-1220.60	-1260.70	-370.7	-590.60	-807.3	-943.20
15	-698.2	-778.10	-547.1	-696.10	-751.2	-734.40	-1329.9	-1260.00	-1274.20	-334.6	-550.90	-767.4	-941.00
16	-683.4	-781.50	-524.7	-656.70	-798.7	-756.20	-1240.5	-1211.90	-1132.40	-318.0	-464.10	-755.6	-912.20
17	-712.6	-725.40	-546.6	-631.30	-727.0	-779.70	-1107.2	-1208.50	-1316.70	-293.8	-442.70	-857.3	-897.60
18	-660.0	-714.60	-579.5	-594.80	-743.2	-760.10	-1182.1	-1143.90	-1294.40	-312.0	-306.70	-816.7	-890.80
19	-648.9	-718.30	-501.1	-607.50	-824.9	-742.40	-1197.8	-1231.60	-1197.10	-300.3	-368.90	-816.3	-892.40
20	-666.5	-736.50	-494.8	-585.10	-717.0	-798.70	-1092.0	-1348.60	-1237.10	-400.6	-327.60	-753.5	-868.30
21	-734.2	-753.00	-504.3	-556.20	-774.1	-836.10	-1277.6	-1155.10	-1152.90	-293.0	-352.80	-832.8	-805.40
22	-636.6	-898.20	-541.8	-558.00	-679.0	-746.10	-1299.7	-1112.00	-1176.60	-233.0	-239.60	-946.4	-844.20
23	-676.1	-735.90	-553.5	-542.30	-702.7	-767.10	-1232.1	-1175.90	-1239.00	-260.8	-318.50	-720.8	-842.80
24	-622.0	-766.20	-553.0	-512.10	-856.3	-701.10	-1093.2	-1215.00	-1182.20	-201.6	-244.40	-707.0	-820.40
25	-621.1	-709.70	-526.7	-556.70	-727.2	-683.90	-1092.8	-1167.70	-1204.60	-204.3	-228.40	-903.6	-889.40
26	-604.7	-681.60	-487.1	-523.40	-724.8	-740.90	-1086.8	-1280.70	-1124.70	-222.9	-185.80	-789.5	-847.20
27	-619.0	-649.80	-463.3	-523.90	-651.5	-676.10	-1108.0	-1128.60	-1208.50	-184.2	-182.10	-816.0	-824.20
28	-638.9	-763.80	-491.8	-526.40	-756.7	-741.40	-1229.9	-1054.80	-1212.50	-182.3	-175.00	-719.1	-885.20
29	-592.5	-670.40	-511.9	-553.60	-630.3	-735.70	-1097.9	-1235.90	-1149.80	-181.0	-176.10	-832.2	-823.00
30	-664.4	-668.50	-505.3	-519.40	-717.8	-712.90	-1172.5	-1087.90	-1135.00	-169.3	-118.80	-705.1	-831.30
∑	**-22740.3**	**-24367.80**	**-20964.5**	**-22446.90**	**-26269.2**	**-26806.20**	**-37293.6**	**-36716.30**	**-36989.70**	**-14402.6**	**-16709.30**	**-26350.4**	**-28865.60**

Solvent Accessible Surface Area (SASA) – GAPDH (B-form)

CPP count on complex	Solvent Accessible Surface Area (SASA) nm^2																
	Cationic Cell Penetrating Peptides					Hydrophobic Cell Penetrating Peptides						Amphipathic Cell Penetrating Peptides					
	Penetratin	HIV-TAT (47–57)	R10	CCMV Gag	SV40	K-FGF	Integrin β3	Hepatitis B	Grb2	HIV-1 gp41	C105Y	Transportan	pVEC	MPG	CADY	sC18	C6
0	80.67	83.68	92.15	84.82	81.12	81.19	81.13	81.12	81.94	80.49	81.38	79.14	78.32	80.69	81.84	85.39	78.98
1	102.36	102.54	112.94	108.40	104.82	97.34	89.75	89.99	90.47	96.11	99.10	101.34	100.35	106.85	106.11	108.06	105.08
2	121.66	122.33	131.84	133.37	126.93	113.72	103.84	100.95	100.13	107.75	114.55	119.15	122.41	133.42	131.98	130.49	123.35
3	137.67	138.40	151.12	153.91	150.51	125.34	115.63	112.71	109.73	121.99	122.54	140.35	142.03	142.18	155.72	148.52	147.07
4	158.00	153.04	169.06	173.30	172.19	131.27	126.12	119.52	116.05	133.86	131.01	153.55	158.85	166.49	169.64	164.49	171.80
5	177.23	170.97	185.59	194.08	193.33	138.44	137.21	125.99	124.52	143.05	141.90	167.73	175.72	188.54	193.72	185.79	197.47
6	190.93	178.46	195.49	207.91	205.55	149.90	139.12	129.23	127.40	150.25	148.37	185.37	191.59	203.81	203.80	199.95	218.38
7	211.72	193.34	214.94	228.57	229.42	162.43	148.28	136.67	138.03	159.40	156.99	197.78	210.87	230.15	227.17	218.07	238.13
8	211.03	194.51	213.09	229.01	230.08	165.59	148.62	138.01	139.80	158.93	162.54	204.67	210.15	226.44	230.29	220.91	244.66
9	224.56	202.93	224.67	242.97	245.82	170.40	153.01	142.19	138.20	168.68	167.29	214.74	221.95	246.69	233.17	231.79	260.67
10	239.99	213.62	238.19	257.81	261.37	178.46	157.03	147.11	143.33	176.09	179.64	235.79	236.77	262.00	248.22	251.41	281.93
11	253.45	229.07	257.83	281.25	285.90	187.46	160.33	157.33	152.57	185.73	196.40	256.04	256.27	280.22	265.17	264.69	298.05
12	272.75	243.66	277.81	301.46	306.84	197.08	172.14	162.00	155.43	199.16	206.49	261.06	272.30	302.01	286.26	286.29	314.87
13	272.18	243.44	276.45	297.58	312.17	195.06	176.64	162.40	157.38	199.69	211.20	283.43	267.62	304.96	290.21	285.14	315.60
14	287.21	259.41	294.90	318.89	330.61	199.18	180.59	164.78	165.61	202.68	227.24	302.17	285.45	325.18	307.55	305.46	339.55
15	301.70	271.48	311.53	341.09	349.69	200.44	180.20	169.23	161.46	211.52	236.26	306.61	297.36	340.63	317.96	318.88	354.83
16	308.97	281.95	325.07	351.80	362.56	201.49	187.20	173.58	165.32	214.33	231.11	314.88	311.76	350.43	323.71	333.61	365.22
17	322.36	292.05	343.61	369.13	382.72	210.55	189.04	176.43	169.47	219.45	235.36	322.55	331.22	362.63	335.72	354.11	384.45
18	331.49	305.66	362.12	392.46	398.69	209.40	187.51	178.94	171.20	225.77	239.66	324.65	337.45	370.98	335.73	371.89	395.80

19	352.59	319.47	378.90	413.18	416.33	212.85	189.11	184.33	179.30	231.21	250.25	333.02	355.73	387.34	345.90	389.61	407.78
20	353.89	318.79	380.58	414.56	420.11	205.43	183.51	184.88	180.72	227.15	250.81	339.21	352.53	377.90	345.75	390.55	400.07
21	363.38	331.63	391.98	434.77	435.61	210.52	190.71	191.69	185.10	234.13	251.50	338.98	366.25	387.25	359.28	400.40	407.27
22	364.19	327.06	394.30	439.58	442.78	214.36	185.64	185.09	183.25	230.03	250.53	355.02	359.28	384.09	344.78	401.82	400.85
23	369.55	332.71	397.39	441.52	449.09	218.86	179.57	183.86	181.64	222.53	254.44	360.81	360.13	384.07	33908	402.25	397.47
24	380.65	342.07	415.30	454.80	467.01	221.82	177.69	189.47	178.53	221.62	254.32	364.68	364.96	381.57	34042	415.82	396.72
25	382.15	348.26	423.87	462.14	474.80	219.19	176.10	188.52	176.55	214.64	252.69	373.88	377.08	372.71	330.72	416.38	395.58
26	397.18	363.61	441.88	484.45	496.47	223.08	178.21	189.75	179.68	218.86	260.05	381.34	390.12	384.95	338.52	427.66	404.82
27	396.18	371.46	446.88	498.79	505.32	223.43	179.78	185.48	177.83	214.32	250.56	380.81	388.98	379.92	341.36	431.49	405.92
28	406.17	383.38	462.05	514.38	520.35	218.77	182.39	183.51	182.12	214.72	248.69	391.43	392.85	385.70	342.44	448.78	416.11
29	418.83	394.66	477.29	533.90	536.81	219.04	182.64	182.88	178.65	212.89	254.68	384.91	404.82	387.91	339.34	465.07	429.21
30	428.12	402.60	491.40	546.42	553.72	222.62	185.81	185.39	182.53	214.75	261.54	395.58	412.29	392.68	350.92	472.82	432.68
